# Supplementary material for: MARC-3, a membrane-associated ubiquitin ligase, is required for fast polyspermy block in Caenorhabditis elegans
Source: Nat Commun. 2024 Jan 26;15:792. doi: 10.1038/s41467-024-44928-6 (PMC10817901; doi:10.1038/s41467-024-44928-6)
Supplement: Supplementary file 1 — Supplementary Information [file 41467_2024_44928_MOESM1_ESM.pdf]

I. Kawasaki, K. Sugiura, T. Sasaki, N. Matsuda, M. Sato, and K. Sato

MARC-3, a membrane-associated ubiquitin ligase, is required for fast polyspermy block in *Caenorhabditis elegans*

Supplementary Information

Supplementary Fig. 1

A

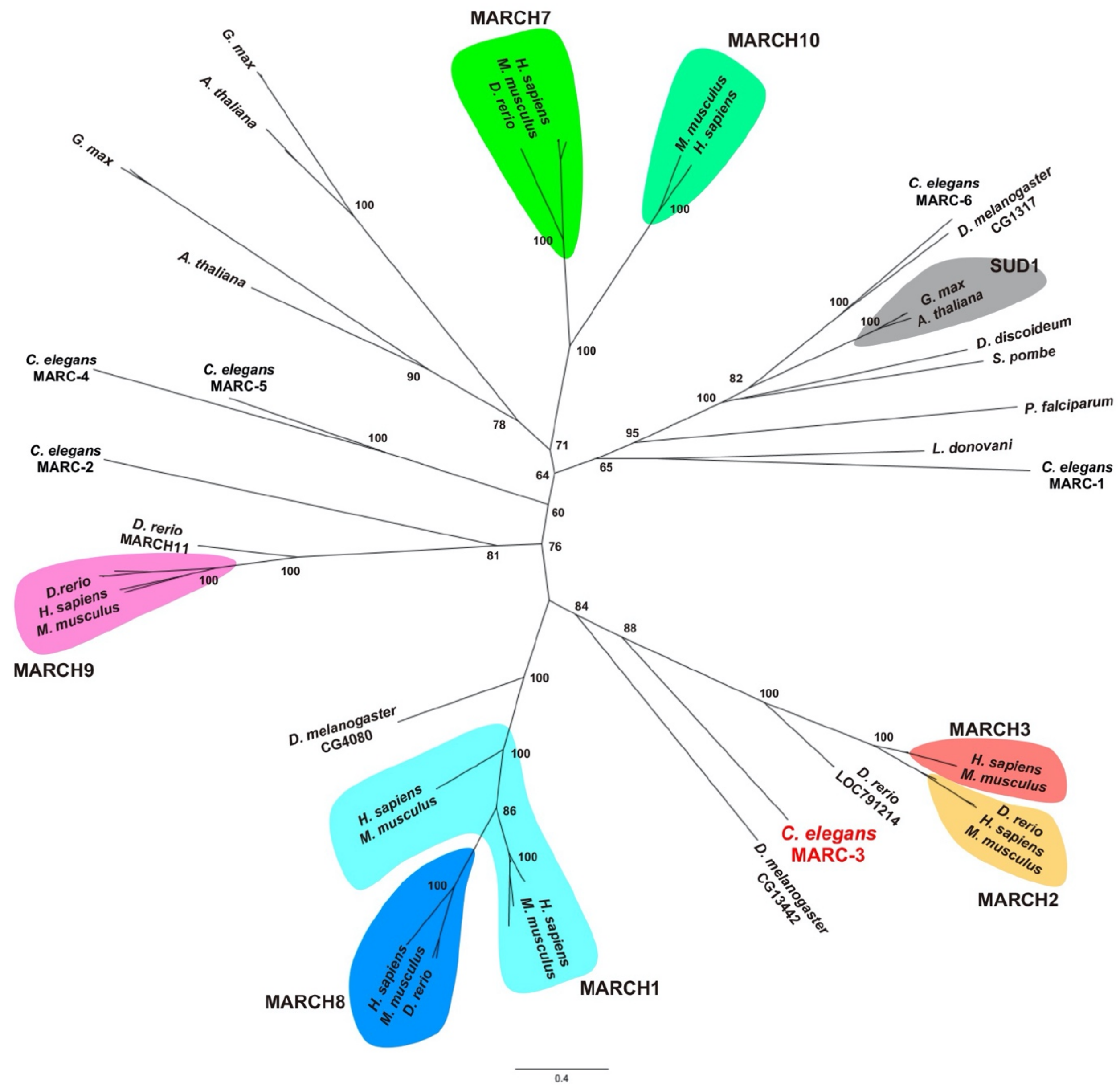

B

```
MARC-4   80 CRICHTST------S-TRSNPLISPCRCSGTLLFVHKACVVRWLEMSTRKMVPSPRCELC 132
MARC-5  325 CRICHCCW------PPDSNDPLISPCRCSGSLQYVHVSCLMHWLDISSRKLHRPAICELC 378
MARC-1   52 CRICQMH-----------EGDMVRPCDCAGTMGDVHEECLTKWVNMSNKK-----TCEIC  95
MARC-6   53 CRVCRGN-----------EGSLYYPCLCTGSIKYVHQECLVEWLKYSKKE-----VCELC  96
MARC-2   25 CRICFDN--------DTSSDSLIKPCSCSGTVAYVHNGCLEQWVRTTSNI-----QCTIC  71
MARC-3   13 CRICMCGETSIPYLGQQAGEPLISPCKCSGTMGLFHRSCLEHWLTLTSTT-----NCEIC  67
            *  *                         *  *        *  *                          *  *
```

Supplementary Fig. 1 (related to Figs. 1 and 7A). Phylogenetic tree of MARCH family proteins in model organisms and multiple alignments of the RING-CH domain of *C. elegans* MARC family proteins

(A) Phylogenetic tree of MARCH family proteins in model organisms drawn using FigTree (http://tree.bio.ed.ac.uk/software/figtree). The number at each node indicates the strength of support for the node calculated using 1000 rapid bootstrap replicates on IQ-TREE [S1]. Scale bar, substitutions per position. The draft data and sequences used on this analysis are available at https://github.com/kentasugiura/kawasakietal_natcomm_rawdata/tree/main/iqtall

(B) CLUSTAL alignment of the RING-CH domain among *C. elegans* MARC family proteins, drawn using the Clustal Omega program at EMBL-EBI (https://www.ebi.ac.uk/Tools/msa/clustalo/).

Supplementary Fig. 2

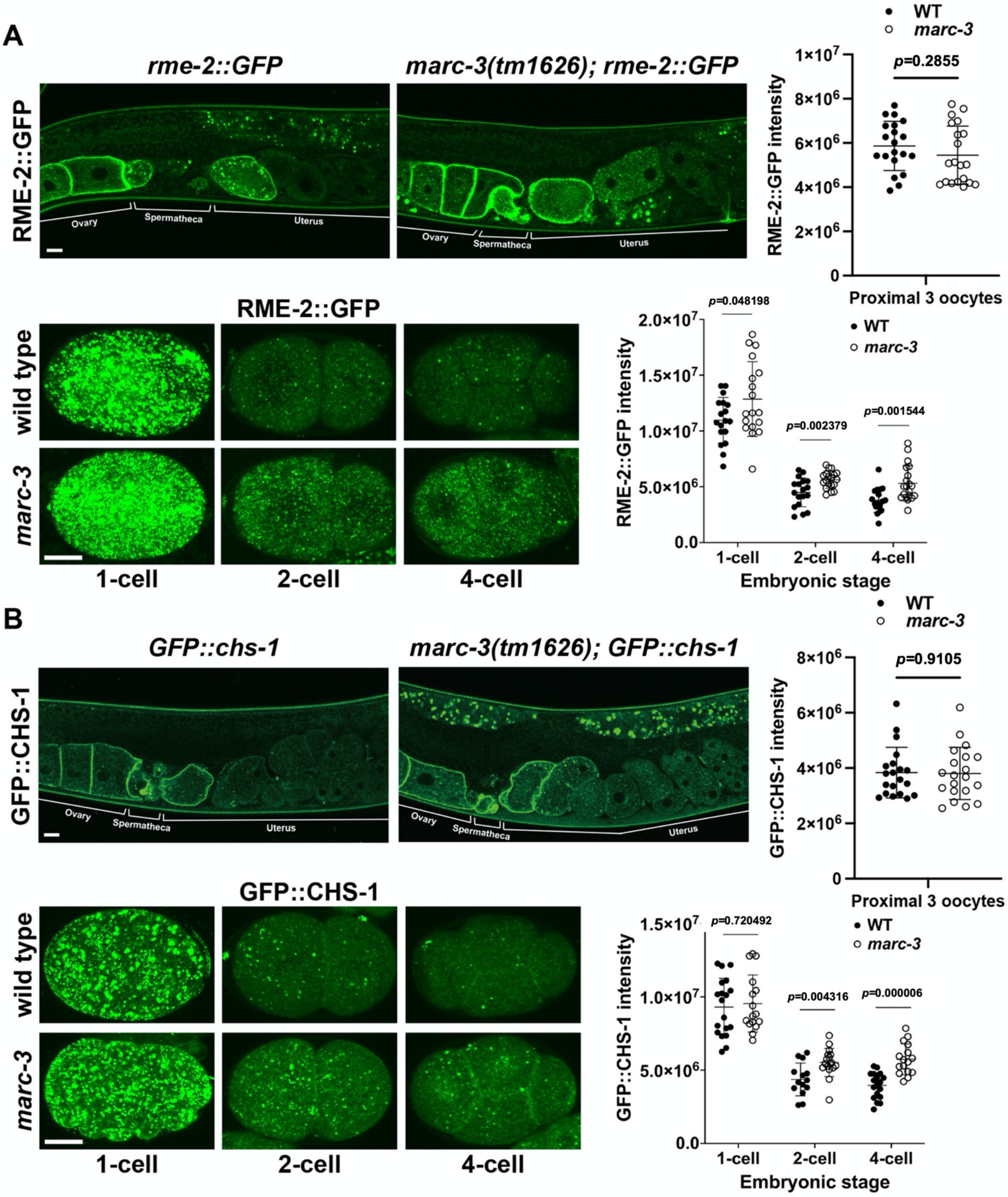

Supplementary Fig. 2. (related to Fig. 1)

MARC-3 is required for the degradation of RME- 2 and CHS-1, two maternal PM proteins

(A) Top half, degradation after fertilization of RME-2::GFP is delayed in *marc-3(tm1626)* mutant adult hermaphrodites (right, n = 18) compared with WT control (left, n = 11). The positions of ovary, spermatheca, and uterus are indicated in the images. The graph on the right shows the distribution of RME-2::GFP intensities obtained from middle focal plane images of proximal three oocytes in wild-type (black circles) and *marc-3(tm1626)* (white circles) adult hermaphrodites (n = 20 for each, $p = 0.2855$). Bottom half, digital quantification of GFP signals in z-stack images of WT and *marc-3* mutant 1-, 2-, and 4-cell-stage embryos expressing RME-2::GFP. Representative z-stack images of WT and *marc-3* mutant embryos at respective stages are shown on the left. Bars, 10 µm. Distribution of RME-2::GFP intensities per embryo at three different stages are shown on the right. n = 18, 18, and 18 for wild type (black), n = 18, 19, and 20 for *marc-3(tm1626)* (white), and $p = 0.048198$, 0.002379, and 0.001544 at the 1-, 2-, and 4-cell stages, respectively.

(B) Top half, degradation after fertilization of GFP::CHS-1 is delayed in *marc-3* mutant adult hermaphrodites (right, n = 12) compared with WT control (left, n = 8). The positions of ovary, spermatheca, and uterus are indicated in the images. The graph on the right shows the distribution of GFP::CHS-1 intensities obtained from middle focal plane images of proximal three oocytes in wild-type (black circles) and *marc-3(tm1626)* (white circles) adult hermaphrodites (n = 20 for each, $p = 0.9105$). Bottom half, digital quantification of GFP signals in z-stack images of WT and *marc-3* mutant 1-, 2-, and 4- cell-stage embryos expressing GFP::CHS-1. Representative z-stack images of embryos at respective stages are shown on the left. Bars, 10 µm. Distribution of GFP::CHS-1 intensities per embryo at three different stages are shown on the right. n = 19, 14, and 18 for wild type (black), n = 16, 17, and 16 for *marc-3(tm1626)* (white), and $p = 0.720492$, 0.004316, and 0.000006 at the 1-, 2-, and 4-cell stages, respectively. Y axis, arbitrary units. P values were calculated using multiple unpaired *t* test (two-tailed). Horizontal lines in each graph indicate mean ± S.D.

Supplementary Fig. 3

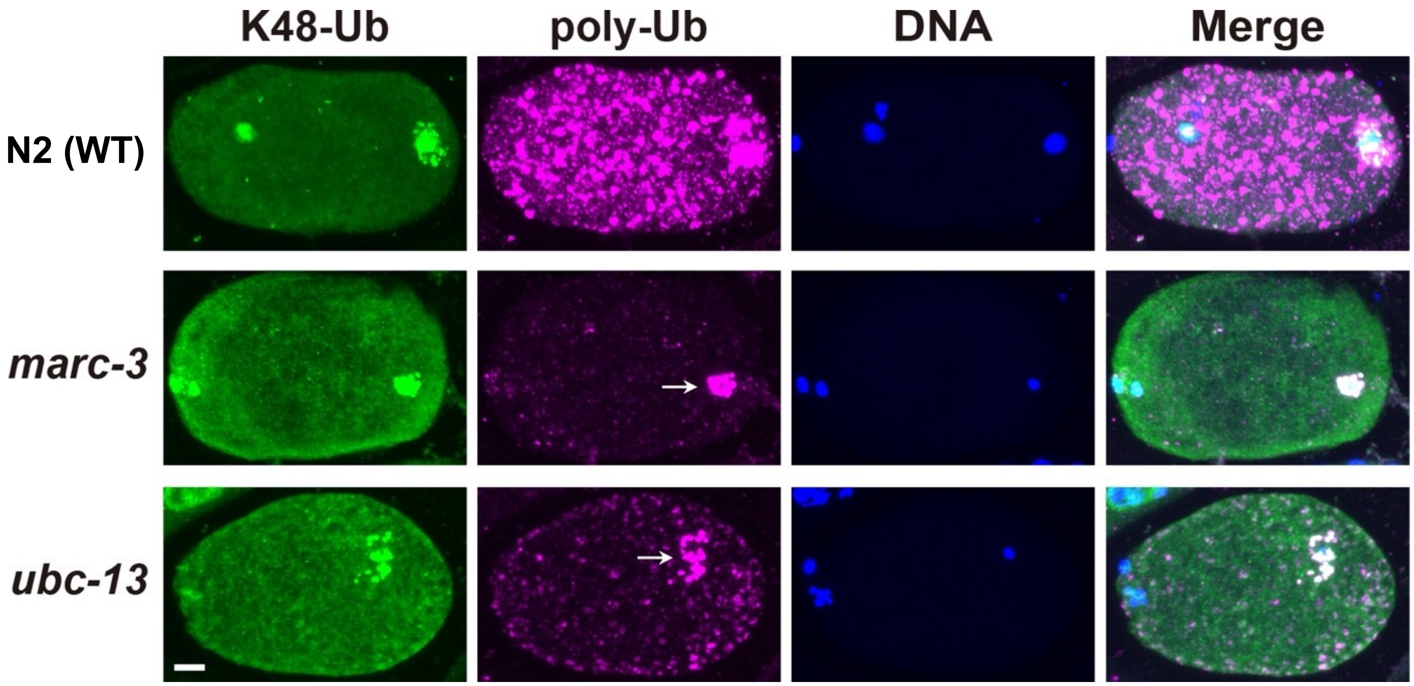

Supplementary Fig. 3 (related to Fig. 2).

K48-linked polyubiquitination was not grossly changed in *marc-3* and *ubc-13* mutant zygotes

Double immunostaining of zygotes of wild-type N2, *marc-3(tm1626)*, and *ubc-13(tm3546)* with anti-K48-linked polyubiquitin antibody (Apu2, green) and anti-polyubiquitin antibody (FK2, magenta). DNA staining, blue. Arrows indicate the ubiquitination of MOs. Bar, 5 µm. Observed embryos, n = 10 for each genotype.

Supplementary Fig. 4

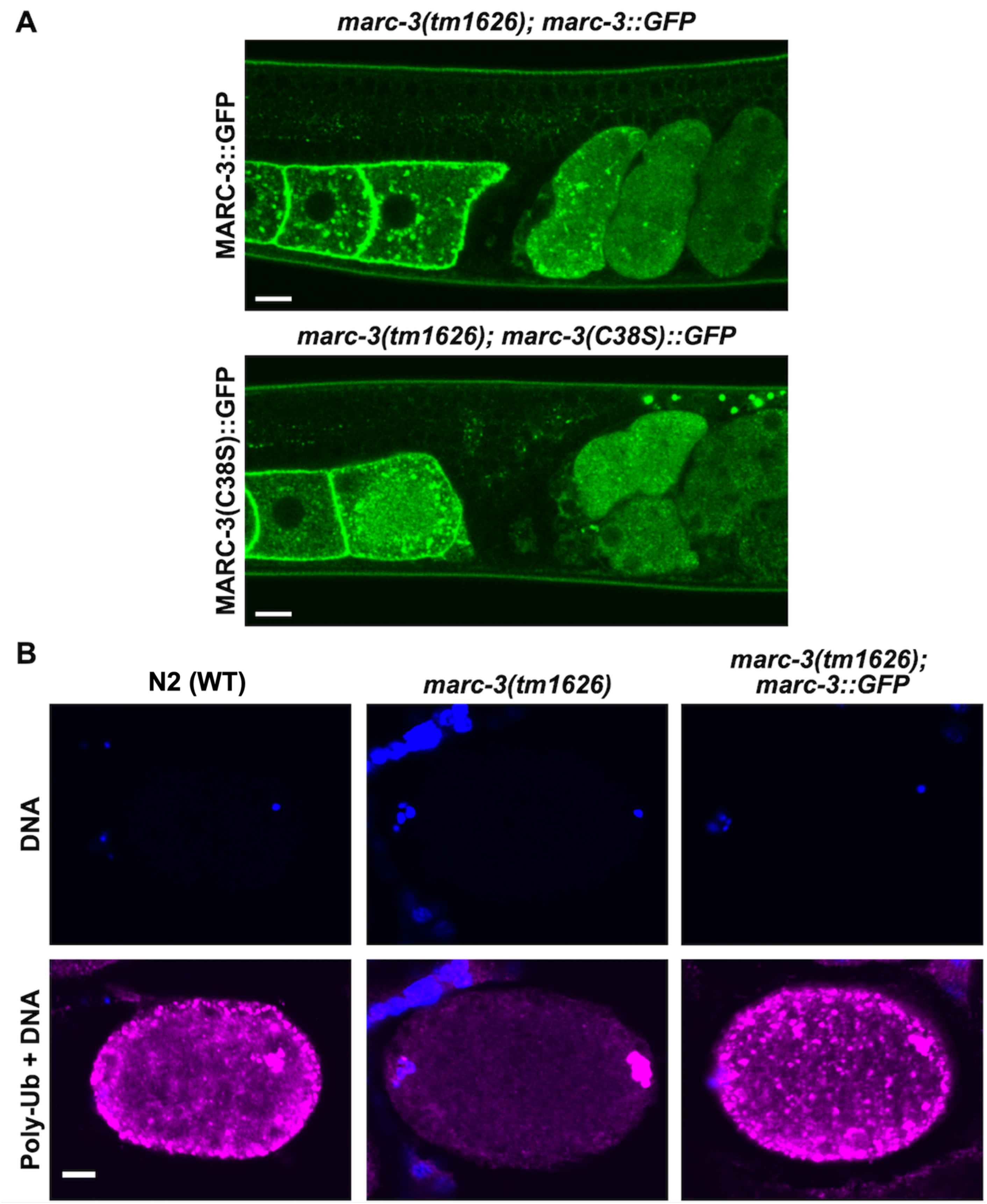

Supplementary Fig. 4 (related to Figs. 3 and 7). The *marc-3::GFP* and the *marc-3(C38S)::GFP* transgenes used in this study

(A) Expression of MARC-3::GFP and its mutant form MARC-3(C38S)::GFP from their transgenes in *marc-3(tm1626)* mutant adult hermaphrodite gonads. In MARC-3(C38S)::GFP, the 38th cysteine was substituted with serine. They showed similar subcellular expression (localization) patterns in late oocytes and early embryos (n = 28 for *marc-3(C38S)::GFP*). However, although *marc-3::GFP* rescued the polyspermy phenotype, *marc-3(C38S)::GFP* failed to rescue the *marc-3(tm1626)* mutant phenotype, as shown in Figure 7C. Bars, 10 µm.

(B) 1-cell-stage embryos of wild-type N2, *marc-3(tm1626)* mutant, and *marc-3(tm1626)* mutant zygotes harboring the *marc-3::GFP* transgene were immunostained with an anti-ubiquitin antibody (FK2, magenta) together with DNA counterstaining (blue). Bar, 5 µm. The *marc-3::GFP* transgene rescued the ubiquitination defect of the *marc-3(tm1626)* mutant (n = 17).

Supplementary Fig. 5

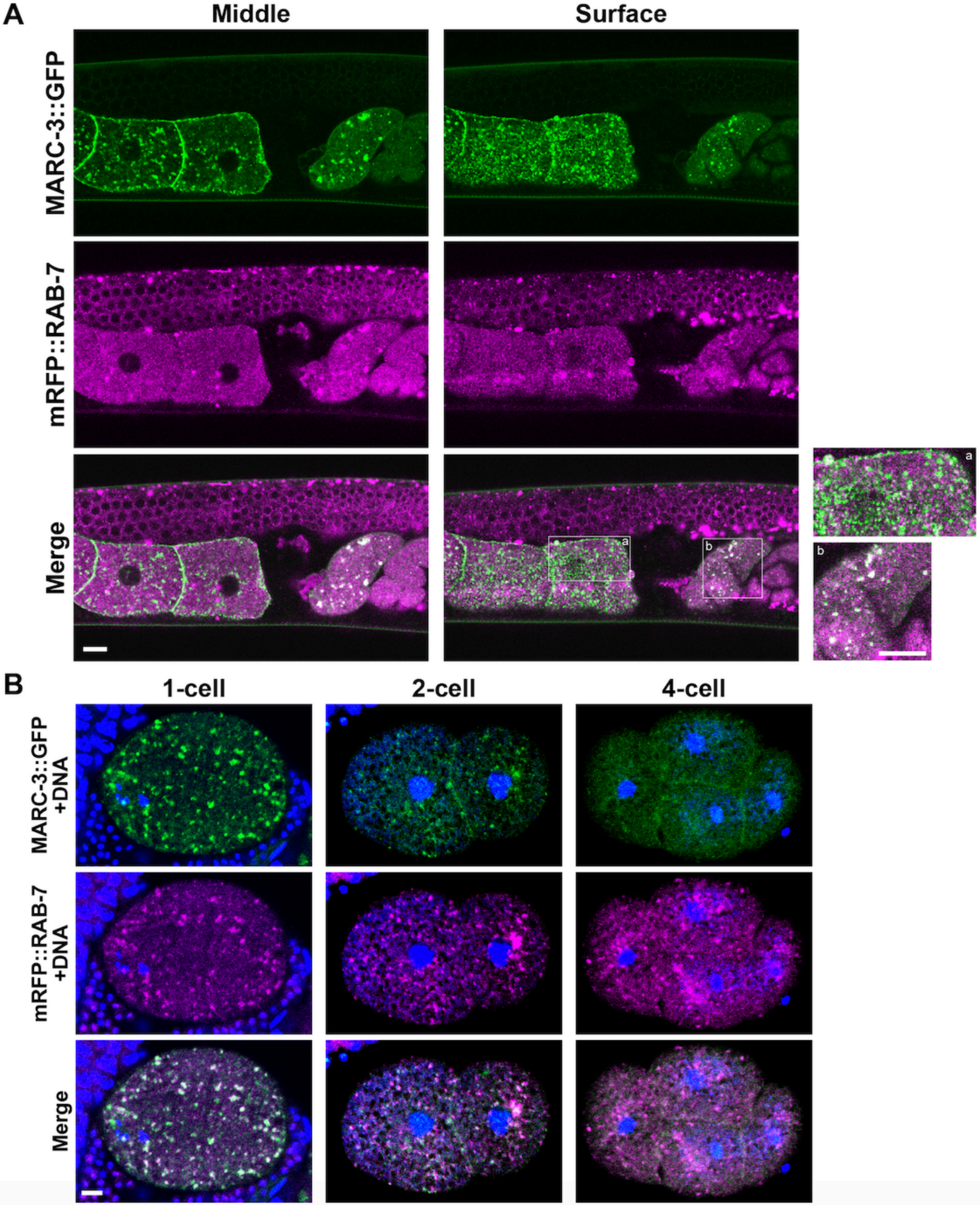

Supplementary Fig. 5 (related to Fig. 3).

Subcellular localization of MARC-3 in oocytes and embryos compared with RAB-7

(A) Subcellular localization of MARC-3::GFP (green) compared with mRFP::RAB-7 (magenta), a late endosome marker, in growing oocytes and early embryos in an adult hermaphrodite gonad. Both middle and surface focal plane images are shown. a and b, enlarged images of boxed areas. Bars, 10 µm. Observed gonads; n = 14.

(B) Subcellular localization of MARC-3::GFP (green) compared with mRFP::RAB-7 (magenta) in 1, 2, and 4-cell-stage embryos. Blue, DNA staining. Bar, 5 µm. Observed zygotes and embryos; n = 13 for zygotes, n = 6 for 2-cell embryos, and n = 6 for 4-cell embryos.

Supplementary Fig. 6

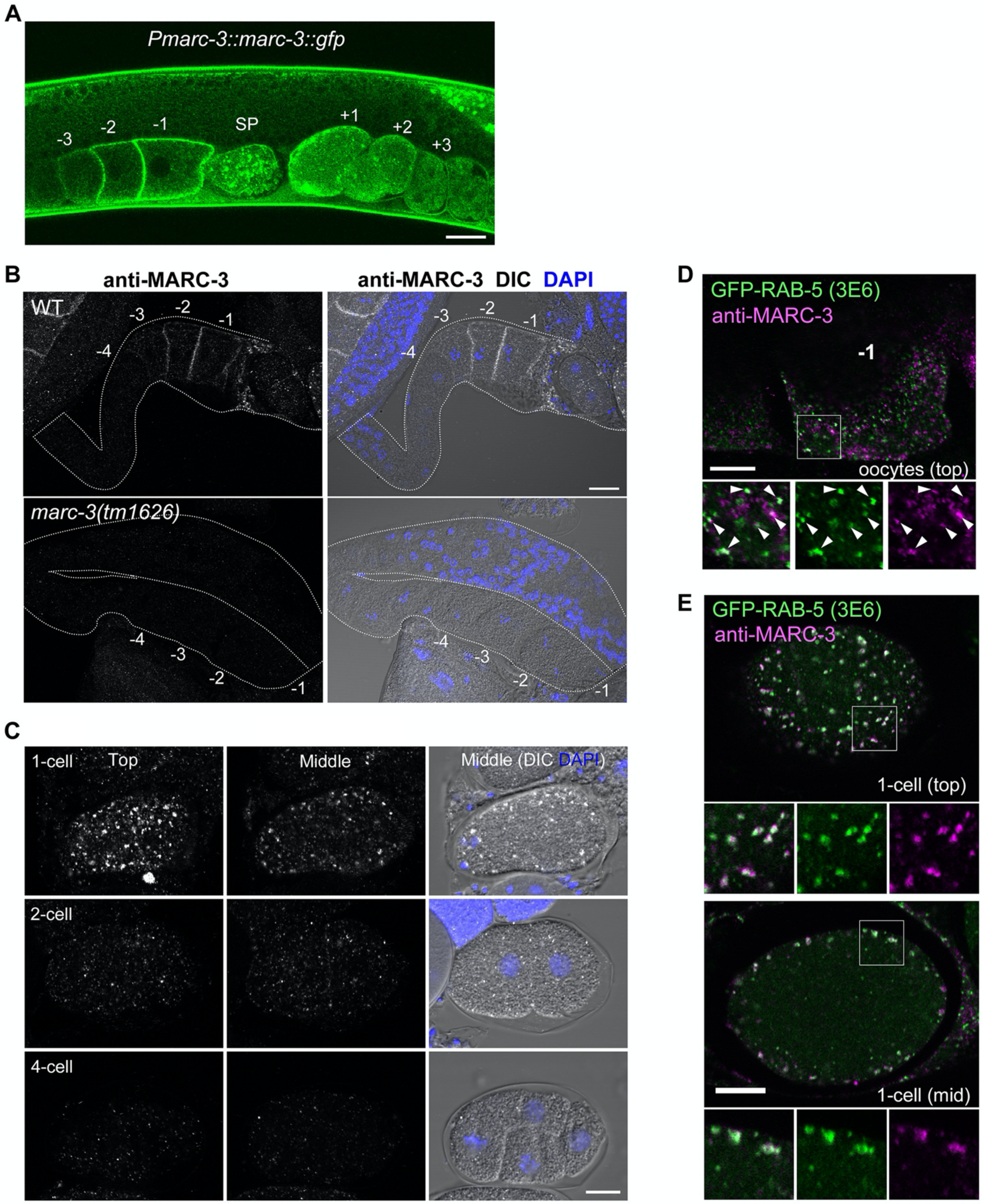

Supplementary Fig. 6 (related to Fig. 3).

Expression of endogenous MARC-3 detected with an endogenously GFP-tagged *marc-3* allele and a MARC-3-specific antibody.

(A) Subcellular localization of MARC-3::GFP expressed from the endogenous *marc-3(syb8421[marc-3::GFP])* allele, which was generated by CRISPR-Cas9 genome editing. Endogenous MARC-3::GFP expressed under its own promoter showed a very similar localization pattern as *pie-1* promotor-driven MARC-3::GFP (n = 24 for *marc-3(syb8421[marc-3::GFP])* animals).

(B) Subcellular localization of endogenous MARC-3 detected with a MARC-3-specific antibody. Gonads were dissected from wild-type and *marc-3(tm1626)* mutant hermaphrodites and immunostained with an anti-MARC-3 antibody (white) and DAPI (blue). MARC-3 was detected in cortical region of growing oocytes. This signal was not detected in the *marc-3* mutant, confirming the specificity of this antibody. n = 21 for wild-type and n = 13 for *marc-3(tm1626)* gonads.

(C) Embryos dissected from wild-type hermaphrodites were immunostained with an anti-MARC-3 antibody and DAPI (blue). MARC-3 was internalized and eliminated from embryos. n = 9 for 1-cell stage and n = 3 for 2- and 4-cell stage embryos. (D and E) Gonads and embryos were dissected from hermaphrodites expressing GFP::RAB-5 and immunostained with anti-MARC-3 (magenta) and anti-GFP (3E6; green) antibodies. MARC-3 partially colocalized with GFP::RAB-5 in oocytes (D; n = 16 gonads). They transiently colocalized on cortical endosomes in zygotes (E; n = 23 zygotes). Bars, 20 µm (A and B) or 10 µm (C-E).

Supplementary Fig. 7

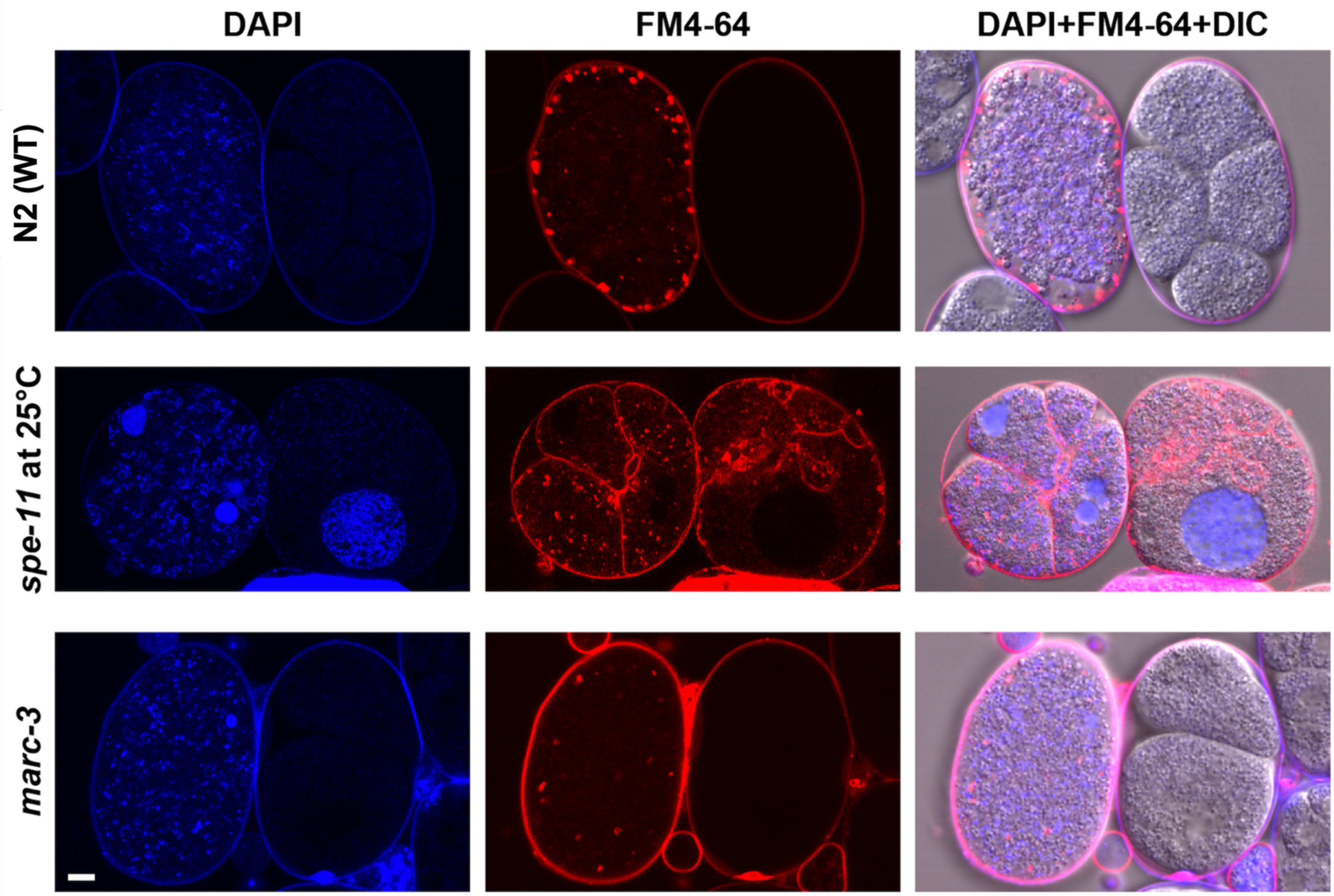

Supplementary Fig. 7 (related to Fig. 5).

Chitin layer formation occurs in *marc-3* mutant embryos

Exposure of unfixed embryos of wild-type N2, *spe-11(hc77)* mutant grown at 25 °C, and *marc- 3(tm1626)* mutant to a solution containing a lipophilic dye, FM4-64 (red), and DAPI (blue). Bar, 5 µm. As N2, the *marc-3* mutant embryos were impermeable to these dyes, except for embryos exposed to these dyes before the completion of metaphase I (n = 14 for N2, n = 24 for *marc-3*).

Supplementary Fig. 8

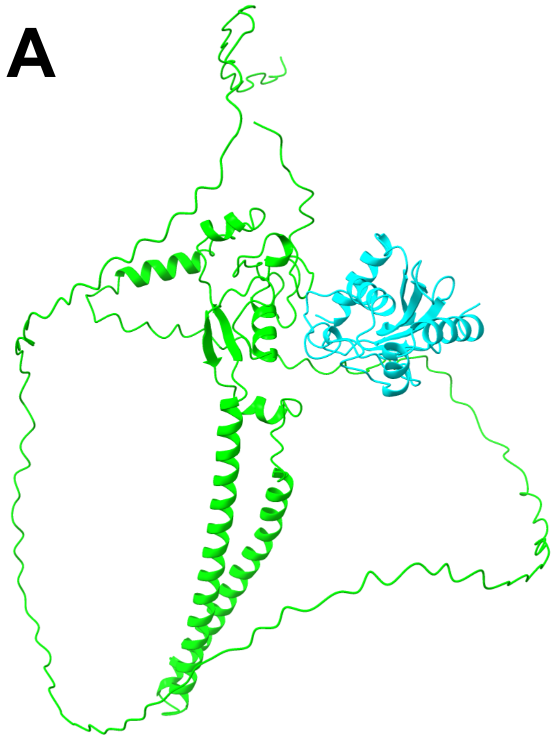

Supplementary Fig. 8 (related to Figs. 4 and 7).

AlphaFold predictions of MARC-3 in complex with LET-70 or EGG-3

(A) AlphaFold2 prediction of MARC-3 (green) in complex with LET-70 (cyan). pLDDT=59.6, pTM=0.514, ipTM=0.861 (pTM+ipTM=1.375).

(B) AlphaFold2 prediction of MARC-3 (green) in complex with EGG-3 (magenta). pLDDT=57.1, pTM=0.544, ipTM=0.442 (pTM+ipTM=0.986).

The draft data and log files are available at https://github.com/kentasugiura/kawasakietal_natcomm_rawdata/tree/main/AF2

Supplementary Fig. 9

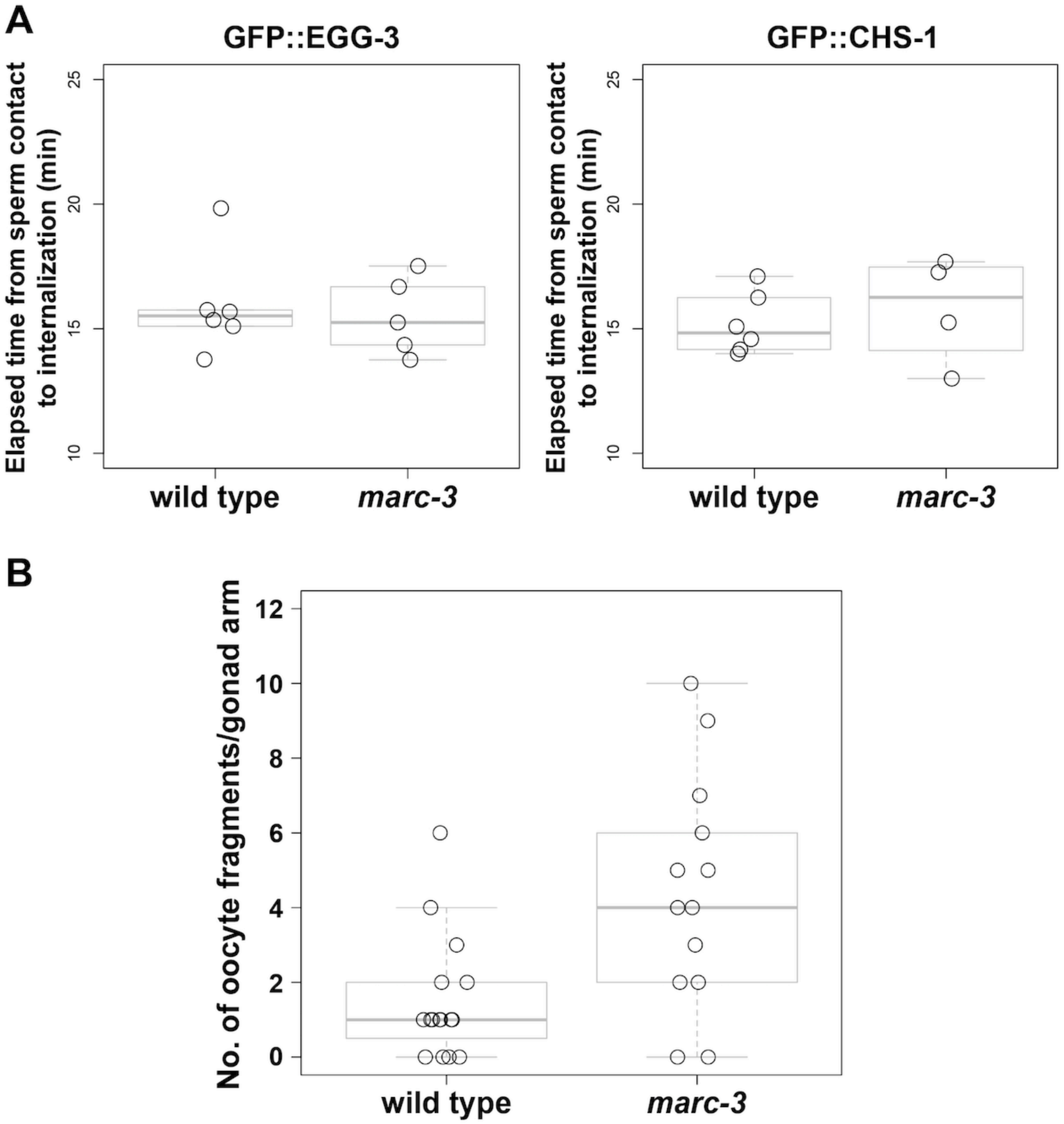

Supplementary Fig. 9 (related to Fig. 7).

MARC-3 may contribute to polyspermy block in mature oocytes before fertilization

(A) Internalization timing of GFP::EGG-3 and GFP::CHS-1 in WT and *marc-3(tm1626)* mutant zygotes. Elapsed time (minutes) from sperm contact to internalization of GFP::EGG-3 (left) and GFP::CHS-1 (right) in WT (n = 6) and *marc-3(tm1626)* mutant zygotes (n = 5 for GFP::EGG- 3, n = 4 for GFP::CHS-1), which were measured using time-lapse live imaging analysis ($p = 0.2286$ and 0.1333, respectively, under Exact Wilcoxon rank sum test).

(B) Distribution of the number of oocyte fragments per gonad arm. n = 16 for WT and n = 13 for *marc-3(tm1626)* mutant. More oocyte fragments were observed in the *marc-3* mutant gonads than in WT gonads during ovulation ($p = 0.009765$ under Exact Wilcoxon rank sum test). Values of the respective points are provided in the Source data file.

Supplementary Fig. 10

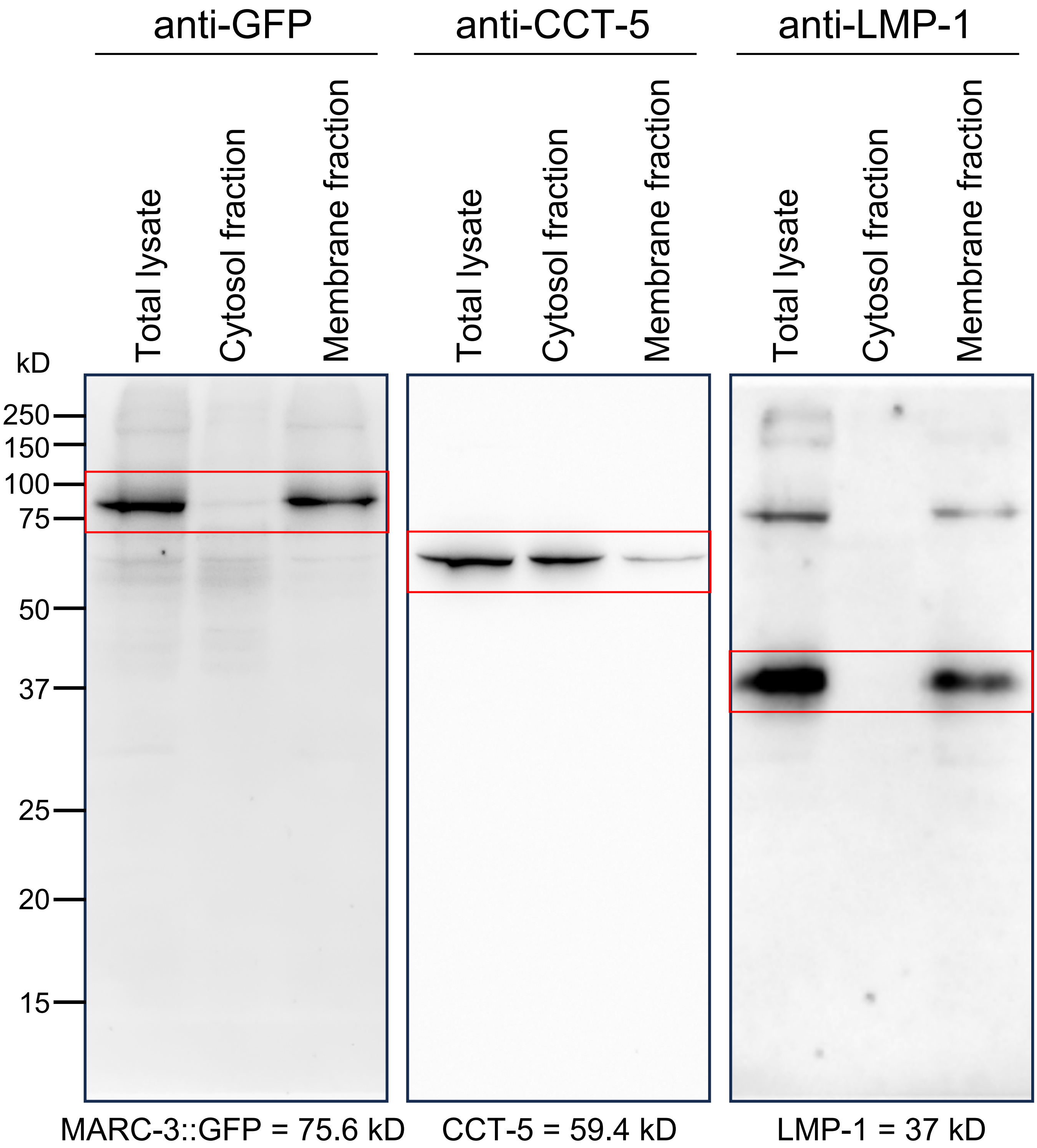

Supplementary Fig. 10 (related to Fig. 3A).

Uncropped original western blot membrane used for Fig. 3A

The membrane was immunoblotted consecutively with anti-GFP, anti-LMP-1, and anti-CCT antibodies. Cropped areas are indicated with red frames. This experiment was repeated two more times (in total, n = 3).

Supplementary Table 1. Brood size and % developmental arrest of *marc-3(tm1626)* mutant

Genotype	Brood size	Unhatched eggs /Mother	Arrested larvae /Mother	% Embryonic lethality	% Larval arrest	% Developmental arrest
N2 (wild type)	307.4 ± 29.0	0 ± 0	0 ± 0	0 (0/3074)	0 (0/3074)	0 (0/3074)
marc-3(tm1626)	248.1 ± 18.1	27.2 ± 11.0	8.7 ± 9.6	11.0 (272/2481)	3.5 (87/2481)	14.5 (359/2481)

Development of self-fertilized progeny were scored at 20ºC. Progeny were produced from 10 hermaphrodites, which were cloned onto separate plates at the L4 larval stage and transferred to new plates every day.

Laid embryos that failed to hatch after 24 hours were judged as ‘unhatched arrested‘ eggs and hatched larvae that remained at the L1 larval stage with uncoordinated movement after the other larvae reached at the L4 larval stage were judged as ‘arrested larvae.’ Numbers are shown as mean ± S.D. (n=10).

Supplementary Table 2. Primer list used in this study

Primer	Sequence
**C17E4.3delF2**	5’-CATTCTGAAGTATTGCAAATTTCCC-3’
**C17E4.3delR1**	5’-CCCGGCATATAAAGCTCCCT-3’
**C17E4.3-GWF2**	5’-GGGGACAACTTTGTACAAAAAAGTTGAAAAAATGGAAGACTTCAACGCATC-3’
**C17E4.3-GWF3**	5'-GGGGACAACTTTGTATTTCCGTCAGTACAA-3’
**C17E4.3-GWR1**	5’-GGGGACAACTTTGTACAAGAAAGTTGTCAAGAATTATAATCATTTT-3’
**C17E4.3-GWR2**	5’-GGGGACAACTTTGTACAAGAAAGTTGTCAGGATCCAGAATTATAATCATTTTCAA-3’
***marc-3N*-GWF3**	5’-GGGGACAACTTTGTACAAAAAAGTTGTGGAAGACTTCAACGCATCGCT-3’
***marc-3N*-GWR3**	5’-GGGGACAACTTTGTACAAGAAAGTTGCTAATTCCGATTTGACTGTAACT-3’
***marc-3*-C38S-F**	5’-ATTTCTCCTTCTAAGTGCTCCGG-3’
***marc-3*-C38S-R**	5’-GAGAGGCTCTCCAGCTTG-3’
**mch3RINGFBamHI**	5’-AAGGATCCGAAGACTTCAACGCATCGCTG-3’
**mch3RINGRPstI**	5’-AAACTGCAGCTAATCAATGAATGGATTCCGATTTG-3’
**mch3C38SF**	5’-CTCTCATTTCTCCTTCTAAGTGCTCCGGAA-3’
**mch3C38SR**	5’-TTCCGGAGCACTTAGAAGGAGAAATGAGAG-3’
***marc-2(syb3694)*IL**	5'-AAAGGTAGAATCGCGTGGAC-3'
***marc-2(syb3694)*IR**	5'-GCATATACACCACCCACGCA-3'
***marc-4* Gtwy-F**	5’-GGGGACAACTTTGTACAAAAAAGTTGTGGCTCAGGACGGAGACCGC-3’
***marc-4* Gtwy-R**	5’-GGGGACAACTTTGTACAAGAAAGTTGCTAGGATGACACGTGGATAC-3’
***marc-5* Gtwy-F2**	5’-GGGGACAACTTTGTACAAAAAAGTTGGCCTGTTCTCGACCGCCTCG-3’
***marc-5* Gtwy-R**	5’-GGGGACAACTTTGTACAAGAAAGTTGCTACTCGAGCACAATCACAG-3’
***ubc-13*-GWF**	5’-GGGGACAACTTTGTACAAAAAAGTTGTGGCCGGGCAACTTCCGCGT-3’
***ubc-13*-R1**	5’-AATTCGCATTCGAATGCACC-3’
***ubc-13*cDNA-GWR**	5’-GGGGACAACTTTGTACAAGAAAGTTGTCAGGCTTGAGCATAGTTCA-3’
***uev-1*-GWF**	5’-GGGGACAACTTTGTACAAAAAAGTTGTGGGTAAGTTCGCTTTCTA-3’
***uev-1*-GWR**	5’-GGGGACAACTTTGTACAAGAAAGTTGTTAGAACATGGCGCCCTCT-3’
***let-70*-GWF**	5’-GGGGACAACTTTGTACAAAAAAGTTGTGGCTCTCAAAAGAATCCAG-3’
***let-70*-GWR**	5’-GGGGACAACTTTGTACAAGAAAGTTGTCACATAGCGTACTTTTGCG-3’
***chs-1*(418-836)-GWF1**	5’-GGGGACAACTTTGTACAAAAAAGTTGCGAGATTGGTTAGACAACGATA-3’
***chs-1*(418-836)-GWR1**	5’-GGGGACAACTTTGTACAAGAAAGTTGTCATTGATAGGCGATGTAGGCAT-3’
***egg-1*(1-48)-GWF1**	5’-GGGGACAACTTTGTACAAAAAAGTTGTGAGCCAGCAACCCGGATCC-3’
***egg-1*(1-48)-GWR1**	5’-GGGGACAACTTTGTACAAGAAAGTTGTCATGACACGCACTTTGCAGCAC-3’
***egg-2*(1-49)-GWF1**	5’-GGGGACAACTTTGTACAAAAAAGTTGTGAGCCAGCAAGCCGGGAAT-3’
***egg-2*(1-49)-GWR1**	5’-GGGGACAACTTTGTACAAGAAAGTTGTCAGGCAACCGGGGGAAACTTTC-3’
***egg-3*-GWF1**	5’-GGGGACAACTTTGTACAAAAAAGTTGTGCGCACCTCTGACAGTCAT-3’
***egg-3*-GWR1**	5’-GGGGACAACTTTGTACAAGAAAGTTGTTAATAAGCCGGTGTGATAC-3’
***egg-4/5*-GWF1**	5’-GGGGACAACTTTGTACAAAAAAGTTGTGGCGTTGAACAGCGAAGTG-3’
***egg-4*-GWR2**	5’-GGGGACAACTTTGTACAAGAAAGTTGTCAGACAGTCGCCACCTTGTTTG-3’
***egg-5*-GWR2**	5’-GGGGACAACTTTGTACAAGAAAGTTGTCACACCTTGTTCGGCATCTCTC-3’
***mbk-2*-GWF1**	5’-GGGGACAACTTTGTACAAAAAAGTTGTGGCTGCTCTTGCCTCGTTC-3’
***mbk-2*-GWR1**	5’-GGGGACAACTTTGTACAAGAAAGTTGTCAGAACTTTTTCCTCATCAAAATATTGGC-3’

Supplementary Table 3. ***C. elegans*** **strain list used in this study**

Strain	Genotype
**N2**	wild type
**NL2098**	*rrf-1(pk1417)I*
**GK866**	*marc-3(tm1626)I*
**PHX5493**	*marc-3(syb5493)I*
**PHX8421**	*marc-3(syb8421[marc-3::GFP])I*
**RT688**	*unc-119(ed3); pwIs281[Ppie-1::cav-1::GFP, unc-119(+)]*
**GK763**	*marc-3(tm1626)I; pwIs281[Ppie-1::cav-1::GFP, unc-119(+)]*
**GK414**	*unc-119(ed3); dkIs241[Ppie-1::GFP::chs-1, unc-119(+)]*
**GK769**	*marc-3(tm1626)I; dkIs241[Ppie-1::GFP::chs-1; unc-119(+)]*
**RT408**	*unc-119(ed3); pwIs116[rme-2:GFP, unc-119(+)]*
**GK770**	*marc-3(tm1626)I; pwIs116[rme-2::GFP; unc-119(+)]*
**PHX3694**	*marc-2(syb3694)II*
**GK1983**	*marc-2(syb3694)II; pwIs281[Ppie-1::cav-1::GFP, unc-119(+)]*
**GK1984**	*marc-3(tm1626)I; marc-2(syb3694)II*
**GK1985**	*marc-3(tm1626)I; marc-2(syb3694)II; pwIs281[Ppie-1::cav-1::GFP]*
**GK1199**	*ubc-13(tm3546)IV*
**GK2017**	*marc-3(tm1626)I; ubc-13(tm3546)IV*
**GK2018**	*marc-3(tm1626)I; ubc-13(tm3546)IV; pwIs281[Ppie-1::cav-1::GFP, unc-119(+)]*
**DLM15**	*ubc-18(tm5426) III*
**RB1976**	*uev-1(ok2610)I*
**GK1078**	*uev-1(ok2610)I; pwIs281[Ppie1::cav-1::GFP, unc-119(+)]*
**GK1220**	*uev-1(ok2610)I; ubc-13(tm3546)IV; pwIs288[Ppie-1::cav-1::GFP, unc-119(+)]*
**GK700**	*unc-119(ed3); dkIs405[Ppie-1::marc-3::GFP]*
**GK2288**	*dkIs405[Ppie-1::marc-3::GFP]; pwIs403[Ppie-1::mCherry::rab-5]*
**GK2165**	*marc-3(tm1626)I; dkIs405[Ppie-1::marc-3::GFP]; pwIs40[Ppie-1::mRFP::rab-7]*
**GK2159**	*dkIs405[Ppie-1::marc-3::GFP]; asIs2[Ppie-1::mCherry::egg-3]*
**GK2458**	*dkIs1092[Ppie-1::marc-3(C38S)::GFP]; asIs2[Ppie-1::mCherry::egg-3]*
**GK2070**	*marc-3(tm1626)I; dkIs405[Ppie-1::marc-3::GFP]*
**GK2424**	*marc-3(tm1626)I; dkIs1092[Ppie-1::marc-3(C38S)::GFP]*
**BA819**	*spe-11(hc77)I*
**GK2200**	*him-5(e1490)V; asIs1[Ppie-1::GFP::egg-3]; dkIs698[Pspe-11::hsp-6::mCherry]*
**GK2206**	*marc-3(tm1626)I; him-5(e1490)V; asIs1[Ppie-1::GFP::egg-3]; dkIs698[Pspe-11::hsp-6::mCherry]*
**GK2119**	*dkIs698[Pspe-11::hsp-6::mCherry]; dkIs241[Ppie-1::GFP::chs-1]*
**GK2136**	*marc-3(tm1626)I; dkIs698[Pspe-11::hsp-6::mCherry]; dkIs241[Ppie-1::GFP::chs-1]*
**GK2231**	*dkIs698[Pspe-11::hsp-6::mCherry]; ltIs38[Ppie-1::GFP::PH(PLCdelta1)]*
**GK2235**	*marc-3(tm1626)I; dkIs698[Pspe-11::hsp-6::mCherry]; ltIs38[Ppie-1::GFP::PH(PLC1 delta1)]*
**AD226**	*egg-3(tm1191)/mIn1[mIs14 dpy-10(e128)]II*
**GK2451**	*marc-3(tm1626)I; egg-3(tm1191)/mIn1[mIs14 dpy-10(e128)]II*
**GK2460**	*egg-3(tm1191)/mIn1[mIs14 dpy-10(e128)]II; dkIs698[Pspe-11::hsp-6::mCherry]*
**RT122**	*unc-119(ed3); pwIs20[Ppie-1::GFP::rab-5)]*
**GK2228**	*asIs2[Ppie-1::mCherry::egg-3]; nnIs2[Ppie-1::GFP::chs-1]*
**GK2229**	*marc-3(tm1626); asIs2[Ppie-1::mCherry::egg-3]; nnIs2[Ppie-1::GFP::chs-1]*

Supplementary References

S1. Trifinopoulos J, Nguyen LT, von Haeseler A, Minh BQ. W-IQ-TREE: a fast online phylogenetic tool for maximum likelihood analysis. *Nucleic Acids Res.* **44**, W232– W235 (2016). doi:10.1093/nar/gkw256.
