# Supplementary material for: MARC-3, a membrane-associated ubiquitin ligase, is required for fast polyspermy block in Caenorhabditis elegans
Source: Nat Commun. 2024 Jan 26;15:792. doi: 10.1038/s41467-024-44928-6 (PMC10817901; doi:10.1038/s41467-024-44928-6)
Supplement: Supplementary file 3 — Description of Additional Supplementary Files [file 41467_2024_44928_MOESM3_ESM.pdf]

Description of Additional Supplementary Files

Supplementary Movie 1 (related to Fig. 3). Time-lapse imaging of MARC-3::GFP and mCherry::RAB-5 in maturing oocytes and early embryos

Time-lapse images of MARC-3:GFP (green) and mCherry::RAB-5 (magenta). Images were obtained every 1.5 s, and the movie was played at 30 frames per second (fps). This movie is related to Fig. 3.

Supplementary Movie 2 (related to Fig. 6B). Time-lapse imaging of HSP-6::mCherry and GFP::PH(PLCδ1) in a marc-3(tm1626) mutant adult hermaphrodite gonad

Time-lapse images of HSP-6::mCherry (magenta) and GFP::PH(PLCδ1) (green) in a marc-3(tm1626) mutant adult hermaphrodite gonad. Images were obtained every 1.5 s, and the movie was played at 30 fps. This movie is related to Fig. 6B.

Supplementary Movie 3 (related to Fig. 6B). Time-lapse imaging of HSP-6::mCherry in an egg-3(tm1191) mutant adult hermaphrodite gonad

Time-lapse images of HSP-6::mCherry (magenta) in an egg-3(tm1191) mutant adult hermaphrodite gonad. Images were obtained every 1.5 s, and the movie was played at 30 fps. This movie is related to Fig. 6B.
