# Supplementary material for: MARC-3, a membrane-associated ubiquitin ligase, is required for fast polyspermy block in Caenorhabditis elegans
Source: Nat Commun. 2024 Jan 26;15:792. doi: 10.1038/s41467-024-44928-6 (PMC10817901; doi:10.1038/s41467-024-44928-6)
Supplement: Supplementary file 7 — Reporting Summary [file 41467_2024_44928_MOESM7_ESM.pdf]

Corresponding author(s): Ken Sato
Last updated by author(s): Dec 21, 2023

Reporting Summary

Nature Portfolio wishes to improve the reproducibility of the work that we publish. This form provides structure for consistency and transparency in reporting. For further information on Nature Portfolio policies, see our Editorial Policies and the Editorial Policy Checklist.

Statistics

For all statistical analyses, confirm that the following items are present in the figure legend, table legend, main text, or Methods section.

n/a	Confirmed	
☐	☒	The exact sample size (n) for each experimental group/condition, given as a discrete number and unit of measurement
☐	☒	A statement on whether measurements were taken from distinct samples or whether the same sample was measured repeatedly
☐	☒	The statistical test(s) used AND whether they are one- or two-sided<br>*Only common tests should be described solely by name; describe more complex techniques in the Methods section.*
☒	☐	A description of all covariates tested
☒	☐	A description of any assumptions or corrections, such as tests of normality and adjustment for multiple comparisons
☐	☒	A full description of the statistical parameters including central tendency (e.g. means) or other basic estimates (e.g. regression coefficient) AND variation (e.g. standard deviation) or associated estimates of uncertainty (e.g. confidence intervals)
☐	☒	For null hypothesis testing, the test statistic (e.g. F, t, r) with confidence intervals, effect sizes, degrees of freedom and P value noted<br>*Give P values as exact values whenever suitable.*
☒	☐	For Bayesian analysis, information on the choice of priors and Markov chain Monte Carlo settings
☒	☐	For hierarchical and complex designs, identification of the appropriate level for tests and full reporting of outcomes
☒	☐	Estimates of effect sizes (e.g. Cohen's d, Pearson's r), indicating how they were calculated

Our web collection on statistics for biologists contains articles on many of the points above.

Software and code

Policy information about availability of computer code

Data collection	ColabFold v1.5.3., IQ-TREE v1.6.12.a, FV10-ASW v4.2.3.6, Micro-Manager v2.0.0., Fusion Solo 7S Edge 18.10
Data analysis	Graphpad Prism 8, Fusion Solo 7S Edge 18.10, R v3.6.1, ChimeraX v1.7.0, ImageJ2 v2.14.0/1.54f, ImageJ v1.53k

For manuscripts utilizing custom algorithms or software that are central to the research but not yet described in published literature, software must be made available to editors and reviewers. We strongly encourage code deposition in a community repository (e.g. GitHub). See the Nature Portfolio guidelines for submitting code & software for further information.

Data

Policy information about availability of data

All manuscripts must include a data availability statement. This statement should provide the following information, where applicable:

- Accession codes, unique identifiers, or web links for publicly available datasets
- A description of any restrictions on data availability
- For clinical datasets or third party data, please ensure that the statement adheres to our policy

The source data have been provided in the Source Data file. No sequence or proteomic data has been generated in this study. All data supporting the findings of this study are available from the corresponding authors upon request. Image files are available upon request because they are many large files in a unique format from the microscope software.

Research involving human participants, their data, or biological material

Policy information about studies with human participants or human data. See also policy information about sex, gender (identity/presentation), and sexual orientation and race, ethnicity and racism.

Reporting on sex and gender	Not involving human participants on this study.
Reporting on race, ethnicity, or other socially relevant groupings	Not involving human participants on this study.
Population characteristics	Not involving human participants on this study.
Recruitment	Not involving human participants on this study.
Ethics oversight	Not involving human participants on this study.

Note that full information on the approval of the study protocol must also be provided in the manuscript.

Field-specific reporting

Please select the one below that is the best fit for your research. If you are not sure, read the appropriate sections before making your selection.

[x] Life sciences [ ] Behavioural & social sciences [ ] Ecological, evolutionary & environmental sciences

For a reference copy of the document with all sections, see nature.com/documents/nr-reporting-summary-flat.pdf

Life sciences study design

All studies must disclose on these points even when the disclosure is negative.

Sample size	Sample sizes for all analyses determined following established protocols and standard practice in C. elegans field.
Data exclusions	No data was excluded.
Replication	We conducted multiple independent experiments to collect data at least twice. The number of experiments was described in the Figure legend. Each experiment reproducibly showed the reported results.
Randomization	No randomization was used.
Blinding	Investigators were not blinded.

Reporting for specific materials, systems and methods

We require information from authors about some types of materials, experimental systems and methods used in many studies. Here, indicate whether each material, system or method listed is relevant to your study. If you are not sure if a list item applies to your research, read the appropriate section before selecting a response.

Materials & experimental systems

n/a	Involved in the study	
[ ]	[x]	Antibodies
[x]	[ ]	Eukaryotic cell lines
[x]	[ ]	Palaeontology and archaeology
[ ]	[x]	Animals and other organisms
[x]	[ ]	Clinical data
[x]	[ ]	Dual use research of concern
[x]	[ ]	Plants

Methods

n/a	Involved in the study	
[x]	[ ]	ChIP-seq
[x]	[ ]	Flow cytometry
[x]	[ ]	MRI-based neuroimaging

Antibodies

Antibodies used	Mouse anti-ubiquitin antibody (FK2; Medical and Biological Laboratories, Nagoya, Japan, Cat. No. D058-3, diluted to 1:10,000)<br>Rabbit anti-K48-linked ubiquitin chain antibodies (Apu2, Millipore, Tokyo, Japan: Cat. No. 05-1307, diluted to 1:1,000)<br>Rabbit anti-K63-linked ubiquitin chain antibodies (Apu3, Millipore, Tokyo, Japan: Cat. No. 05-1308, diluted to 1:200)<br>Mouse monoclonal anti-MO antibody (1CB4, a gift from Dr. S. L'Hernault, diluted to 1:1,000)<br>Mouse anti-GFP monoclonal antibody (3E6, Life Technologies, Cat. No. A-11120, dilution 1:100)

	Rabbit anti-MARC-3 polyclonal antibody (This study, diluted to 1:50~100)<br>Alexa Fluor 488 goat anti-mouse IgG (Life Technologies, Cat. No. A11029, Lot No. 2066710, dilution 1:1,000)<br>Alexa Fluor 555 goat anti-mouse IgG (Life Technologies, Cat. No. A21424, Lot No. 2480093, dilution 1:1,000)<br>Alexa Fluor 488 goat anti-rabbit IgG (Life Technologies, Cat. No. A11034, dilution 1:1000)<br>Alexa Fluor 555 goat anti-rabbit IgG (Life Technologies, Cat. No. A21429, Lot No. 1445240, dilution 1:1,000)<br>Goat anti-GFP antibody (Fitzgerald Industries International, Cat. No. 70R-GG001, Lot No. X12090612, dilution 1:2,000)<br>Rabbit anti-CCT-5 polyclonal antibody (Saegusa et al., 2014, affinity purified, dilution 1:500)<br>Mouse monoclonal anti-LMP-1 antibody (DSHB, Iowa City, IA, USA, dilution 1:500)<br>Donkey anti-goat IgG, HRP conjugate (Millipore, Cat. No. AP180P, Lot No. NG1899148, dilution 1:5,000)<br>Peroxidase-conjugated goat anti-rabbit IgG (Jackson ImmunoResearch, Cat. No. 111-035-003, Lot No. 113298, dilution 1:5,000)<br>Peroxidase-conjugated goat anti-mouse IgG (Jackson ImmunoResearch, Cat. No. 115-035-003, Lot No. 94781, dilution 1:5,000)<br>Rabbit Anti-MBP polyclonal antibody (New England Biolabs, Cat. No. E8030S, dilution 1:4,000)<br>Mouse anti-ubiquitin monoclonal antibody (P4D1, Santa Cruz Biotechnology, sc-8017, dilution 1:500)<br>Goat anti-rabbit IgG-Alkaline Phosphatase (Santa Cruz Biotechnology, sc-2007, dilution 1:5,000)<br>Goat anti-mouse IgG-Alkaline Phosphatase (Santa Cruz Biotechnology, sc-2008, dilution 1:10,000)<br>FM4-64 (Invitrogen, Cat. No. F34653, Lot No. 2103475, used at 1 mM)<br>Rhodamine-conjugated Chitin-binding probe (New England BioLabs, Cat. No. P5210S, Lot No. 4, dilution 1:500)
Validation	We generated a rabbit anti-MARC-3 polyclonal antibody and checked its reactivity against endogenous MARC-3 by immuostaining of wild-type and marc-3 KO animals. This antibody showed some non-specific signals in somatic spermathecal cells. A mouse monoclonal anti-MO antibody 1CB4, was obtained from Dr. S. L'Hernault, who checked its reactivity against membranous organelles (MOs) in C. elegans (Okamoto and Thomson, 1985). Specificity of Rabbit anti-CCT-5 polyclonal antibody was checked in our previous paper (Saegusa et al., 2014). The other antibodies were commercially available and their specificities were checked by each company or institute.

Animals and other research organisms

Policy information about studies involving animals; ARRIVE guidelines recommended for reporting animal research, and Sex and Gender in Research

Laboratory animals	Organism: Caenorhabditis elegans.<br>All strains we used in this study are listed in Supplementary Table 3.<br>Stage: Whole life (from zygote, embryo, L1, L2, L3, L4, and adult).<br>We basically used young adult hermaphrodites to observe oocyte growth, oocyte maturation, fertilization and embryogenesis.
Wild animals	No wild animal was included.
Reporting on sex	Most experiments were carried out using hermaphrodites. Males were used for mating assay.
Field-collected samples	No field-collected sample was included.
Ethics oversight	No ethics oversight is needed in C. elegans study.

Note that full information on the approval of the study protocol must also be provided in the manuscript.

Plants

Seed stocks	No plant was used.
Novel plant genotypes	No plant was used.
Authentication	No plant was used.
